# Reversible Cyclic Thermal Inactivation of Oligopeptidase B from Serratia proteamaculans

**Published:** 2018

**Authors:** M. V. Ovchinnikova, A. G. Mikhailova, D. M. Karlinsky, V. A. Gorlenko, L. D. Rumsh

**Affiliations:** Shemyakin–Ovchinnikov Institute of Bioorganic Chemistry, Russian Academy of Sciences, Miklukho-Maklaya Str. 16/10, Moscow, 117997, Russia; Moscow State Pedagogical University, M. Pirogovskaya Str. 1, bldg. 1, Moscow, 119991, Russia

**Keywords:** oligopeptidase B, Serratia proteamaculans, thermal inactivation

## Abstract

A unique property was found for oligopeptidase B from *Serratia proteamaculans *(PSP) as well as its mutants: they can undergo reversible thermal inactivation at 37°C, with activity being restored or even increased with respect to the initial one upon subsequent cooling. The process can be repeated several times, with the same results achieved (up to 5 cycles). This effect can be explained by a shift in the equilibrium between the inactive open form of the enzyme and the active closed one upon variation of the incubation temperature.

## INTRODUCTION

Oligopeptidase B (OpdB) [EC 3.4.21.83] is a trypsin-like serine peptidase belonging to the prolyl oligopeptidase family. OpdB is present in unicellular eukaryotes, such as trypanosomes *Trypanosoma cruzi* [1], *T. brucei* [2], and *T. evansi* [3], and in leishmania spp. (*Leishmania major* and *L. amazonensis* [4]). OpdB or the genes encoding this enzyme have been detected in prokaryotes, such as *Escherichia coli* [5], *Moraxella lacunata* [6], *Salmonella enterica *serovar *typhimurium* [7], *Yersinia pestis* [7], *Serratia marcescens*, *Stenotrophomonas maltophilia* and *Rhodococcus erythropolis* [8], in mycobacteria *Mycobacterium tuberculosis* and *M. leprae* [7], and in spirochetes *Treponema denticola *[9]. Members of the oligopeptidase B group are also found in some higher plants (e.g., *Ambrosia artemisiifolia* [10]). To date, protozoan OpdB have been the most extensively studied, and the crystal structures of OpdB from *L. major *[11] and *T. brucei *were determined in [12]. Neither the crystal structure nor the enzymological characteristics of most bacterial oligopeptidases B have been determined; only the nucleotide sequences of the genes coding for them are known.

Our study focused on oligopeptidase B from *Serratia proteamaculans *(PSP). The *OpdB S. proteamaculans *94 gene has been cloned, sequenced, and expressed in *E. coli*; the substrate specificity of OpdB, inhibition, and effect of calcium ions, pH, and temperature on enzyme activity have been studied [13-18].

All the previously investigated oligopeptidases B, both protozoan and bacterial, are characterized by high thermal stability [5, 19]. PSP is the first known psychrophilic oligopeptidase B. This enzyme is rather quickly inactivated at 37°C; thermal inactivation is independent of buffer nature and occurs identically in the phosphate, imidazole, and Tris-buffers at pH 7.5– 8.0 [17]. The intrinsic fluorescence spectra demonstrate that heating at 37°C forces the PSP molecule to unfold, which is accompanied by a reduction in enzyme activity. Calcium ions accelerate and enhance PSP inactivation [17].

When experimentally studying thermal inactivation of PSP, Mikhailova et al. [17] revealed that enzyme activity was restored after some enzyme samples had been incubated at low temperatures. In this work, we thoroughly investigated this phenomenon at [Ca^2+^] = 0 and 50 mM.

## EXPERIMENTAL

The reagents used were Nα-benzoyl-*D,L*-arginine *p*-nitroanilide (BAPNA) (Sigma, USA); Tris and NaCl (Merck, Germany), glycerol (ICN, USA); dimethyl sulfoxide (DMSO), and *p*’-guanidine benzoic acid *p*-nitrophenyl ester (Fluka, Germany).

Wild-type PSP and point mutants expressed in *E. coli *BL21(DE3) (Novagen) were obtained and purified according to the procedure described in [17, 20].

Optical density was measured on an Eppendorf BioSpectrometer®kinetic spectrophotometer (Germany). Protein concentration was determined by the Bradford protein assay using the Bio-Rad Protein Assay reagent, with BSA used as a reference protein. Molarity of the enzyme solutions was measured by titrating active sites with *p*’-guanidine benzoic acid *p*-nitrophenyl ester [21].

**Activity of the PSP samples **was studied spectrophotometrically at 25°C, using BAPNA (0.2 mM) as a substrate, in 0.1 M Tris-HCl buffer, pH 8.0, containing 50 mM CaCl_2_ and 2% DMSO. The increase in optical density at 405 nm, which took place as soon as free *p*-nitroaniline (Δε_405_ = 10400 M^–1^cm^–1^) had formed, was measured. The initial hydrolysis rates of the substrate (two or three replicas for each reading; the rates differed by no more than 5–10%) were determined from the initial linear section of the kinetic curve (the degree of hydrolysis was ≤ 10%).

**Investigation of the thermal stability of PSP samples **The initial activities of the wild-type enzyme and its mutant variants (0.05 mg/ml = 0.65 μM) were determined by diluting the stock solution of the enzyme heated to 25°C in the incubation buffer at the same temperature, collecting 5–10 μl aliquots, and measuring the initial hydrolysis rate of BAPNA substrate (0.2 mM; total volume, 1.5 ml). The aliquots of the enzyme solutions (100 μl; 0.65 μM) were incubated for a corresponding time at a corresponding temperature: 5–10 μl aliquots were collected in a quartz cell containing BAPNA, and residual activity was measured immediately according to the procedure described above. The control samples of all PSP variants with the same concentration (0.05 mg/ml = 0.65 μM) were incubated at 25 and 4°C for a corresponding time according to the same procedure as the one used for the experimental specimens (except for heating), and their activity was determined.

## RESULTS AND DISCUSSION

A typical feature of the enzymes belonging to the prolyl oligopeptidase family, including OpdB, is that they contain the N-terminal β-propeller domain, which prevents penetration of bulky globular proteins into the active site, and the catalytic domain residing in the C-terminal portion of the molecule. Studies focused on the crystal structures of the enzymes belonging to the prolyl oligopeptidase family demonstrated that these proteins exist in the open (inactive) and closed (active) forms, which exist in equilibrium. The identified crystal structures of protozoan OpdB from *L. major *[11] and *T. brucei *[12] showed that the function of these enzymes depends not only on the amino acid residues comprising the catalytic triad in its active site and the primary substrate-binding site, but also on five inter-domain salt bridges – SB1–SB5 – that involve nine charged amino acid residues. The salt bridge SB1 – E172/179-R650/664 (trypanosomes/leishmania spp.) plays a crucial role: the catalytic triad is either formed or destroyed as the bridge closes or opens when the molecule conformation changes from the open inactive form to the closed active one, respectively, and vice versa. The salt bridges SB1–SB5 are strictly conserved in all protozoan OpdB.

The amino acid sequence of PSP is 35% homologous with respect to those of OpdB from *L. major *and *T. brucei*; the degree of homology is higher in the C-terminal catalytic domain (50%). Meanwhile, the regions of the active site and the primary substrate-binding site in all these enzymes are virtually identical. It turned out, however, that the five functionally important inter-domain salt bridges detected in the protozoan enzymes are not conserved. Only one of them (SB3) is found in PSP and in other known bacterial OpdB. In PSP and the other investigated bacterial OpdB, either uncharged or oppositely charged amino acid residues occupy positions corresponding to five out of the seven charged residues of OpdB from *L. major *and *T. brucei*, which form the salt bridges SB1, SB2, SB4, and SB5. The key salt bridge responsible for enzyme activity is also absent.

In order to elucidate the mechanism of action of PSP, we simulated the crystal structure of the protein in its closed and open forms [20].

As a result, we revealed 12 charged residues forming the structure consisting of inter- and intra-domain salt bridges that are responsible for the structure and activity of PSP. Eight of those (E75, E96, E125, D647, D649, K655, R658, and K660) were replaced with uncharged residues by site-directed mutagenesis. The corresponding mutant enzymes were obtained and characterized; the residues listed above were shown to play an important role. Removal of charged a.a.r. 75, 96, 655, and 658 resulted in enzyme inactivation, while its activity was enhanced after a.a.r. 125 and 649 had been removed. Depending on the substrate used, substitution of a.a.r. 647 and 660 resulted in either a 2- to 3-fold reduction or a 1.5–2 increase in enzyme activity [20].

In this study, we compared the thermal stability of wild-type PSP (wt), four point mutants (D647A, D649A, K655A, and K660A; the charged residues in the His652-loop of PSP being replaced with uncharged ones) and two point mutants (E75A and E125A; the acidic residues localized in the N-terminal β-propeller domain being replaced with uncharged ones) at [Ca^2+^] = 0 and 50 mM.

As we have repeatedly demonstrated earlier, activity of wild-type PSP samples [15, 17, 18] and its mutant variants [20] in 0.1 Tris-HCl buffer, pH 8.0, at *T *≤ 25 °C remains virtually unchanged for an appreciably long time (up to 10–14 days) irrespective of whether Ca^2+^ ions are absent or present (50 mM). In this study, activity of the initial PSP samples stored at both 4°C and 25°C also remained constant during the entire experiment.

**Fig. 1:**
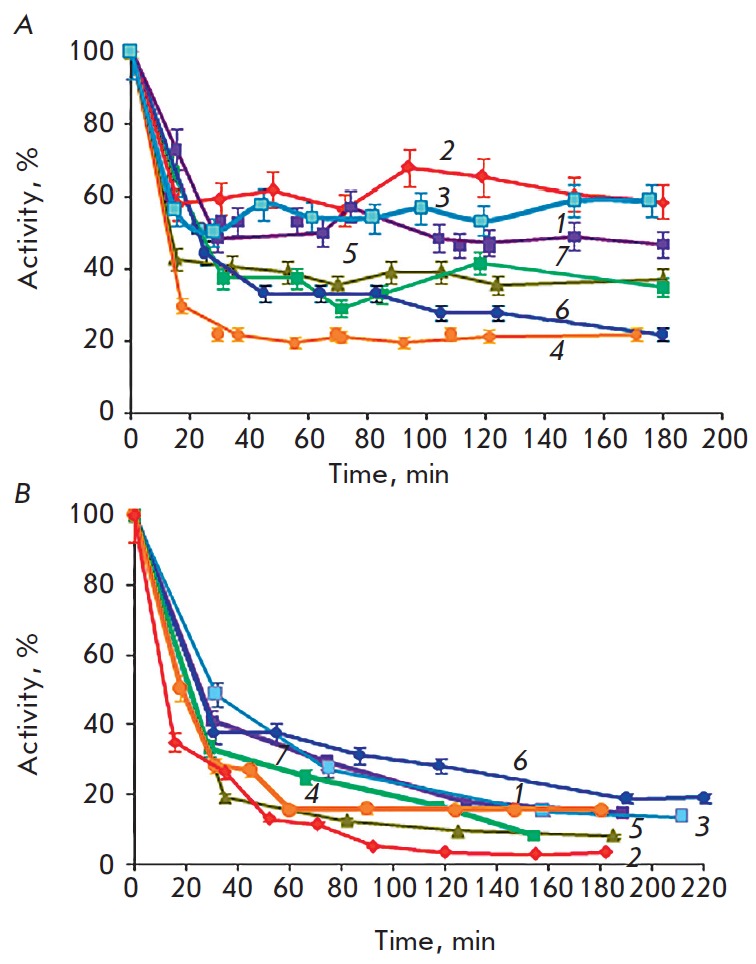
Inactivation of PSP (wt) and the mutant variants of the enzyme (0.65 μM) during incubation at 37°C (0.1 M Tris-HCl buffer pH 8.0; BAPNA substrate) A – [Ca^2+^] = 0; B – [Ca^2+^] = 50 mM; 1 – wt; 2– E75A; 3 – E125A; 4 – D647A; 5 –D649A; 6 – K655A; and 7 – K660A. Initial activity (*v*_0_/[E], min^-1^): wt – 739; E75A – 200; E125A – 1846; D647A – 346; D649A – 923; K655A – 300; and K660A – 323.

The data shown in *Fig. 1 A,B* illustrate that calcium ions are a destabilizing factor for all PSP variants. Thermal stability of the E75A and E125A mutants at [Ca^2+^] = 0 is 20–25 % higher than that of the wild-type enzyme, while the thermal stability of D649A, K655A, K660A and especially D647A mutants is 1.5–2-fold lower (*Fig. 1A*). At [Ca^2+^] = 50 mM, the difference in the thermal stability of all PSP variants was less marked, but the E75A mutant was the most thermally labile one (*Fig. 1B*).

**Fig. 2:**
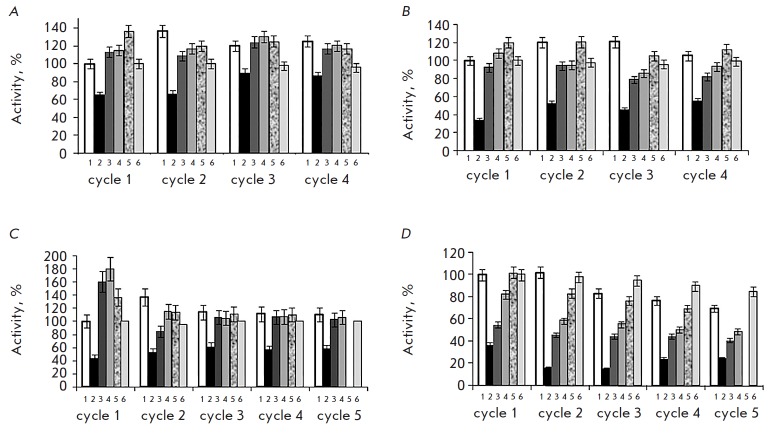
Influence of the cyclic heating/cooling on the activity of mutant PSP variants (0.65 μM) in 0.1 M Tris-HCl pH 8.0; A – wt; B –E125A; C – E75A; and D – D649A. 1 – initial activity; 2 – 37°C; 3 h; 3, 4 – 25°C; 0.5 and 1 h, respectively; 5 – 4°C; 18–20 h; 6 – control: 25°C; 4 h and 4°C; 18–20 h. Initial activity (*v*_0_/[E], min^-1^): wt – 739; E75A – 200; E125A – 1846; and D649A – 923.

According to the data on the inactivation of PSP and the corresponding mutant variants of this enzyme shown in *Fig. 1*, we chose to perform incubation at 37°C for 3 h to study the reactivation of PSP variants. After determining the residual activity, enzyme samples were incubated at 25°C (0.5 and 1 h) and their activities were determined; the samples were then left at 4°C for 18–20 h. The activities of the wild-type enzyme and all the mutant variants were found to increase (*Fig. 2 A–D*); for the wt, E125A, and E75A variants; a 1.2– 1.8-fold increase in the initial activity of OpdB was systematically observed upon cooling of the partially thermally inactivated sample (*Fig. 2 A–C*). Upon cooling, the activity of D649A was as high as 100–107% (*Fig. *2*D*).

It is even more interesting that these heating–cooling cycles of an aliquot of PSP variants can be repeated (up to 5 times); each cycle involves a drop in activity observed upon heating (37°C) and a further increase upon cooling (25 and 4°C). Upon subsequent cycles (2–5), the restored activity exceeded the initial one to a lesser extent or was hardly higher; the activity of the D649A variants in cycles 4 and 5 after cooling was lower than its initial one (~80%) (*Fig. 2D*). Similar experiments on the reactivation of the D647A, K655A, and K660A variants also revealed that activity increased as the partially denatured enzyme was cooled; however, reactivation in the first two cycles did not exceed 75–80% of the initial activity of the samples or 45–50% in the subsequent cycles (data are not presented).

**Fig. 3:**
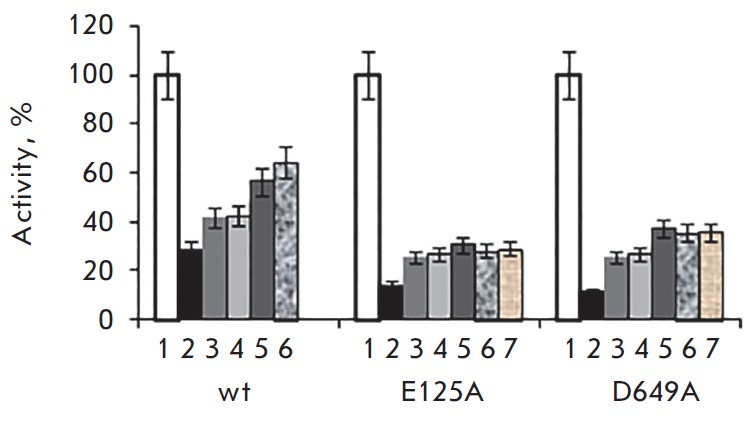
Influence of heating at 37 °C and subsequent cooling on the activity of PSP (wt), E125A and D649A (0.65) in 0.1 M Tris-HCl, pH 8.0, containing 50 mM CaCl_2_. 1 – initial activity; 2 – 37°C; 3 h; 3, 4 – 25°C; 0.5 and 1 h, respectively; 5, 6, 7 – 4°C; 18–20, 36–48 and 54–72 h, respectively.

Cooling the partially inactivated samples in the presence of calcium ions also led to an increase in activity; however, the activity was lower than 100% of the initial value even after long-term incubation (for several days) at 4°C: 64% (wt), 36% (D649A), and 28% (E125A) (*Fig. 3*).

We had previously studied the thermal stability of a PSP molecule by high-sensitivity differential scanning calorimetry (HS-DSC); the results were indicative of a low thermal stability for PSP. The heat capacity curve for the protein featured two peaks with *T*d = 43.1°C (corresponding to the less stable C-terminal catalytic domain) and 46.3°C (the more stable N-terminal β-propeller) [17].

**Fig. 4:**
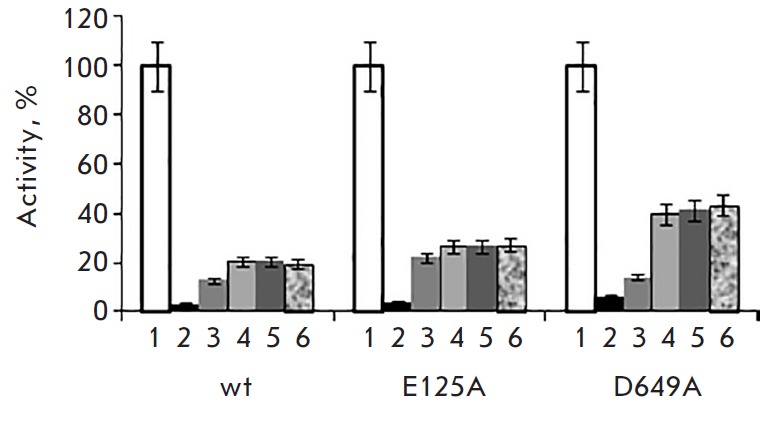
Influence of heating at 43 °C and subsequent cooling on the activity of PSP (wt), E125A and D649A (0.65) in 0.1 M Tris-HCl pH 8.0. 1 – initial activity; 2 – 43°C; 0.5 h; 3, 4 – 25°C; 0.5 and 1 h, respectively; 5, 6, – 4°C; 18–20 and 36–48 h, respectively.

We studied the effect of incubation at 43°C (the denaturation temperature of the C-terminal catalytic domain) for 0.5 h, followed by cooling at 37°C according to the experimental scheme, on the activity of PSP (wt, E125A, and D649A). The residual activity after heating was a percentage of the initial value; nevertheless, sequential incubation at 25 and 4°C increased the activity of PSP variants by an order of magnitude (up to 20–40%) (*Fig. 4*).

Incubation of wt, E125A, and D649A for 0.5 at 46°C (the temperature corresponding to the melting temperature of the N-terminal β-propeller domain) fully inactivated the enzyme; subsequent incubation resulted in protein reactivation at neither 25 nor 4°C (data not presented).

## CONCLUSIONS

**Fig. 5:**
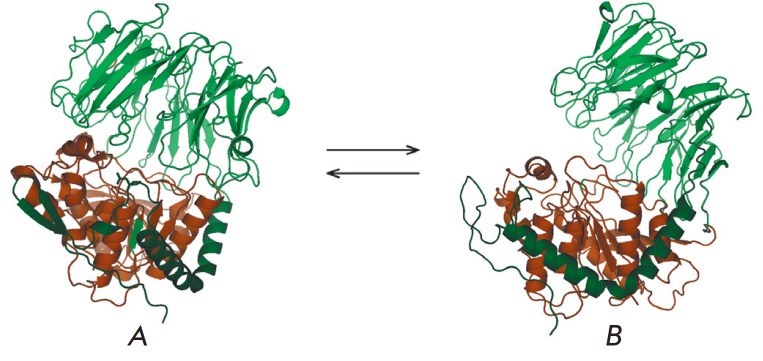
Active closed (*A*) and inactive open (*B*) PSP structures. N-terminal peptide 1–80 is shown in dark green; N-terminal β-propeller domain 81-408 is shown in green; the C-terminal catalytic domain 409–677 is shown in brown.

A unique property of oligopeptidase B from *S. proteamaculans *(PSP) has been revealed: it can undergo reversible thermal inactivation at 37°C, while subsequent cooling results in recovery of, or even an increase in, enzyme activity. The process can be repeated several times (up to five cycles), with the same results. This effect can be attributed to the fact that heating shifts the equilibrium between the inactive open enzyme form and the active closed one towards the open form. Subsequent cooling causes a reverse shift towards the closed active form. This hypothesis relies on the results of X-ray diffraction and NMR studies of the nature of reversible transitions between different forms of enzymes of the prolyl oligopeptidase family [12] and our earlier findings about the correlation between the thermal inactivation of PSP at 37°C and unfolding of its protein molecule (according to the intrinsic fluorescence spectra) [17]. *Figure 5* shows the closed and open forms of PSP corresponding to an earlier obtained model of the enzyme [20]. It is clear that the inactive open form is an unfolded molecule (i.e., a denatured enzyme).

It is more difficult to explain the simultaneously occurring rise in activity. The intermediate, more active forms of the enzyme may form during the transition. Indeed, Canning et al. [12] conducted NMR studies of human prolyl oligopeptidase to find out that numerous conformations of this protein exist in solution, suggesting that the molecules of the enzymes of this family, including OpdB, are in constant movement and sequentially adopt a number of different conformations, including the completely open and completely closed ones.

Calcium ions impede the reverse transition to the closed form. The reduction in the thermal stability of PSP in the presence of Ca^2+^ can be attributed to the destruction of the salt bridges SB2 and SB3 that takes place as Ca^2+^ binds to the E494 and D460 residues partaking in bridge formation. Indeed, substitution of the corresponding charged amino acid residues for the uncharged ones in OpdB from *T. brucei *significantly reduced the thermal stability of these mutants. A conclusion has been drawn [22] that the salt bridges SB2 and SB3 play a structural role in and are responsible for the stability of the OpdB molecule.

## Abbreviations

PSP: protease from Serratia proteamaculans
OpdB: oligopeptidase B
BAPNA: Nα-benzoyl-D, L-arginine p-nitroanilide
DMSO: dimethyl sulfoxide

## References

[1] Burleigh B.A., Caler E.V., Webster P., Andrews N.W. (1997). J. Cell Biol..

[2] Morty R.E., Shih A.Y., Fülöp V., Andrews N.W. (2005). FEBS Lett..

[3] Morty R.E., Pelle R., Vadasz I., Uzcanga G.L., Seeger W., Bubis J.J. (2005). J. Biol. Chem..

[4] de Matos Guedes H.L., Duarte Carneiro M.P., de Oliveira Gomes D.C., Rossi-Bergmann B., Giovanni De-Simone S. (2007). Parasitol. Res..

[5] Yan J.B., Wang G.Q., Du P., Zhu D.X., Wang M.W., Jiang X.Y. (2006). Prot. Expr. Purif..

[6] Yoshimoto T., Tabira J., Kabashima T., Inoue S., Ito K. (1995). J. Biochem..

[7] Morty R.E., Fülöp V., Andrews N.W. (2002). Journal of Bacteriology.

[8] Mustafa M.S.M., Nakajima Y., Oyama H., Iwata N., Ito K. (2012). Biol. Pharm. Bull..

[9] Fenno J.C., Lee S.Y., Bayer C.H., Ning Y. (2001). Infect. Immun..

[10] Bagarozzi D.A. Jr., Potempa J., Travis J. (1998). Am. J. Respir. Cell Mol. Biol..

[11] McLuskey K., Paterson N.G., Bland N.D., Isaacs N.W., Mottram J.C. (2010). J. Biol. Chem..

[12] Canning P., Rea D., Morty R.E., Fulop V. (2013). PloS One..

[13] Mikhailova A.G., Likhareva V.V., Khairullin R.F., Lubenets N.L., Rumsh L.D., Demidyuk I.V., Kostrov S.V. (2006). Biochemistry (Moscow)..

[14] Khairullin R.F., Mikhailova A.G., Sebyakina T.Yu., Lubenets N.L., Ziganshin R.Kh., Demidyuk I.V., Gromova T.Yu., Kostrov S.V., Rumsh L.D. (2009). Biochemistry (Moscow)..

[15] Mikhailova A.G., Khairullin R.F., Demidyuk I.V., Gromova T.Yu., Kostrov S.V., Rumsh L.D. (2011). Biochemistry (Moscow)..

[16] Mikhailova A.G., Khairullin R.F., Kolomijtseva G.Ya., Rumsh L.D. (2012). Biochemistry (Moscow)..

[17] Mikhailova A.G., Khairullin R.F., Demidyuk I.V., Kostrov S.V., Grinberg N.V., Burova T.V., Grinberg V.Ya., Rumsh L.D. (2014). Protein Exp. Purif..

[18] Mikhailova A.G., Nekrasov A.N., Zinchenko A.A., Rakitina T.V., Korzhenevsky D.A., Lipkin A.V., Razguljaeva O.A., Ovchinnikova M.V., Gorlenko V.A., Rumsh L.D. (2015). Biochemistry (Moscow)..

[19] Ismail N.I.M., Yuasa T., Yuasa K., Nambu Y., Nisimoto M., Goto M., Matsuki H., Inoue M., Nagahama M., Tsuji A. (2010). J. Biochem..

[20] Mikhailova A.G., Rakitina T.V., Timofeev V.I., Karlinsky D.M., Korzhenevsky D.A., Agapova Yu.K., Vlaskina A.V., Ovchinnikova M.V., Gorlenko V.A., Rumsh L.D. (2017). Biochimie..

[21] Walsh K.F., Wilcox P.E. (1970). Meth. Enzymol..

[22] Fukumoto J., Ismaliza N., Ismail M., Kubo M., Kinoshita K., Inoue M., Yuasa K., Nishimoto M., Matsuki H., Tsuji A. (2013). J. Biochem..

